# Impact of a community-based perinatal and newborn preventive care package on perinatal and neonatal mortality in a remote mountainous district in Northern Pakistan

**DOI:** 10.1186/s12884-015-0538-8

**Published:** 2015-04-30

**Authors:** Zahid A Memon, Gul N Khan, Sajid B Soofi, Imam Y Baig, Zulfiqar A Bhutta

**Affiliations:** Greenstar Social Marketing, Karachi, Pakistan; Department of Paediatrics and Child Health, Aga Khan University, Karachi, Pakistan; Aga Khan Health Services, Northern Areas, Gilgit-Baltistan, Pakistan; Centre of Excellence in Women and Child Health, Aga Khan University, Karachi, Pakistan; Center for Global Child Health, Hospital for Sick Children, Toronto, Canada

**Keywords:** Perinatal, Neonatal, Newborn, Mortality, Community-based, Package, Gilgit

## Abstract

**Background:**

There is limited evidence from community-based interventions to guide the development of effective maternal, perinatal and newborn care practices and services in developing countries. We evaluated the impact of a low-cost package of community-based interventions implemented through government sector lady health workers (LHWs) and community health workers (CHWs) of a NGO namely Aga Khan Health Services on perinatal and neonatal outcomes in a sub-population of the remote mountainous district of Gilgit, Northern Pakistan.

**Methods:**

The package was evaluated using quasi experimental design included promotion of antenatal care, adequate nutrition, skilled delivery and healthy newborn care practices. Control areas continued to receive the routine standard health services. The intervention areas received intervention package in addition to the routine standard health services. Outcome measures included changes in maternal and newborn-care practices and perinatal and neonatal mortality rates between the intervention and control areas.

**Results:**

The intervention was implemented in a population of 283324 over a 18 months period. 3200 pregnant women received the intervention. Significant improvements in antenatal care (92% vs 76%, p < .001), TT vaccination (67% vs 47%, p < .001), institutional delivery (85% vs 71%, p < .001), cord application (51% vs 71%, p < .001), delayed bathing (15% vs 43%, p < .001), colostrum administration (83% vs 64%, p < .001), and initiation of breastfeeding within 1 hour after birth (55% vs 40%, p < .001) were seen in intervention areas compared with control areas. Our results indicate significant reductions in mortality rates in intervention areas as compared to control areas from baseline in perinatal mortality rate (from 47.1 to 35.3 per 1000 births, OR 0.62; 95% CI: 0.56-0.69; P 0.02) and neonatal mortality rates (from 26.0 to 22.8 per 1000 live births, 0.58; 95% CI: 0.48-0.68; P 0.03).

**Conclusions:**

The implementation of a set of low cost community-based intervention package within the health system settings in a mountainous region of Pakistan was found to be both feasible and beneficial. The interventions had a significant impact in reduction of the burden of perinatal and neonatal mortality.

**Trial registration:**

This study is registered, ClinicalTrial.gov NCT02412293.

## Background

Under-five mortality has fallen globally from 12.6 million deaths in 1990 to 6.6 million deaths in 2012 [1]. The share of neonatal mortality among under-five deaths increased from 37% in 1990 to about 44% in 2012, because of a slower decline in the neonatal mortality rate compared to deaths in older children [2]. In Pakistan, over 60% of deaths under 5 years occur during the neonatal period (55 per 1000 live births) and have not changed over the past 6 years [3]. These national averages mask considerable disparities between provinces and districts. Remote districts of the northern mountainous regions and southern areas of the country have the highest perinatal and neonatal mortality rates [4].

Despite some progress in improving perinatal and neonatal mortality through community-based interventions of maternal and neonatal care packages [5,6], relatively few large-scale community-based projects have delivered neonatal interventions within local health systems and through public sector health workers with perinatal and neonatal mortality as a defined outcomes [7-11].

Available evidence suggests that promotion of maternal and newborn care practices through implementation of community-based packages, including promotion of essential newborn care and community mobilization, are effective in improving neonatal survival in low income settings [12-15]. Nevertheless fewer studies have evaluated impact of community based interventions in remote mountainous regions with limited access. Despite the existence of strong primary care programs in the Northern mountainous regions of Pakistan there has been no evaluation of a community-based strategy to address perinatal and newborn care.

We hypothesized that a coordinated community-based program of community education and awareness creation delivered through primary health care workers will lead to improved maternal and newborn care practices, and reduction in perinatal and neonatal mortality in intervention areas compared to control areas in district Gilgit, Gilgit-Baltistan province of Pakistan.

## Methods

### Study area and population

Gilgit is one of the seven districts and capital city of Gilgit-Baltistan province of Pakistan situated amid the Hindu Kush, Karakoram, and Himalayan mountain ranges. The area is situated about 600 kilometers away from Islamabad, the capital of Pakistan. The population of district Gilgit is around 283,324, majority of which are subsistence farmers [16]. The health infrastructure comprised of three Basic Health Units (BHUs), one Rural Health Centre (RHC), five Civil Hospitals and one District Head Quarter Hospital. In addition to these public sector health facilities, the district is also served by health facilities and outreach workers deployed by Aga Khan Health Services, a non-government organization (NGO). Overall the health services are provided through small and medium sized health centers rather than big hospitals.

### Study design

**Figure 1:**
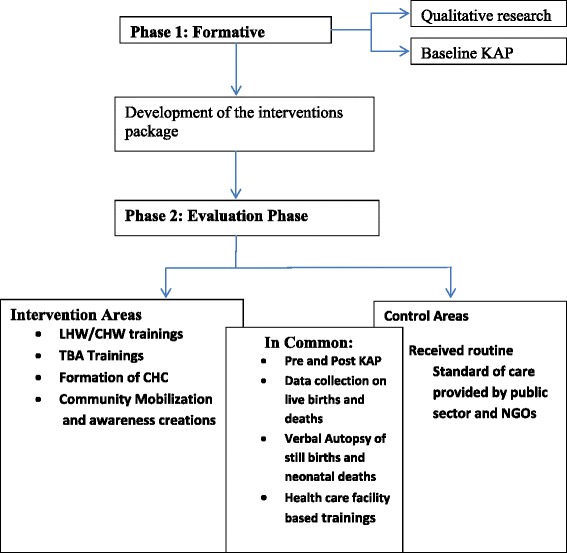
Study phases.

The study followed an exploratory quasi-experimental design. The project was carried out in two distinct phases of formative phase and implementation phase (Figure 1).

### Phase I: formative research

The implementation of any program for improving perinatal and newborn care must be based on a complete assessment and analysis of determinants of perinatal and neonatal morbidity and mortality. While some data on this exist in Pakistan, it was important to collect information from the research site for designing a culturally acceptable intervention package relevant to the local context.

The formative research was conducted from September, 2002 to February, 2003. Forty-eight villages were randomly selected for this phase. The objectives of the formative research were three fold 1) Assess the perinatal and newborn care practices at household level 2) Identify the underlying determinants of the practices and beliefs attached to these practices 3) Understand the local community acceptability and cultural feasibility of the interventions within the local health system settings. The findings of the formative research informed the design and delivery platforms of intervention package such as Lady Health Workers (LHWs) and Community Health Workers (CHWs) deployed by Aga Khan Health Services Pakistan (AKHSP). Government of Pakistan introduced community based National Primary Health Care and family Planning Program (known as LHW program) in 1994. As part of this program, the Lady Health Workers (LHW), local resident women with 8 grade of formal education were trained for 18 months and deployed to deliver community based care. The Community Health Workers (CHWs) are local female health workers trained by AKHSP to deliver community level health programs.

### Phase II: implementation phase

**Figure 2:**
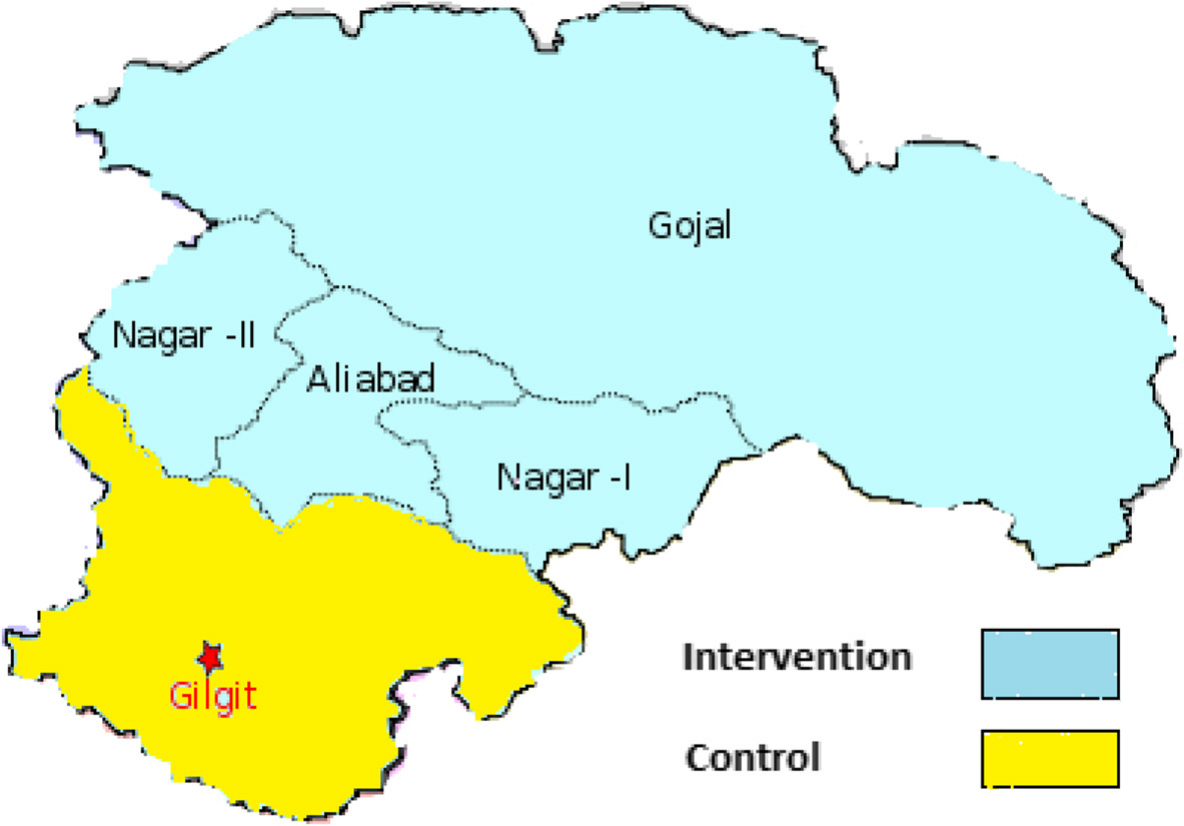
Study district with intervention and control areas.

The objective of phase II was to evaluate the impact of the intervention package on perinatal and neonatal mortality and maternal and newborn care practices using quasi experimental design. The overall population of 283,324 comprising 35,641 households located in the study district was allocated to intervention and control areas based on geographical proximity to avoid contamination and manage logistics and undertake the study with limited resources available (Figure 2). The intervention area was comprised of 16,802 households and population of 137781 and the control area covered 18659 households and population of 145543 (Table 1). The phase II was carried out in sixteen months.

**Table 1:** **Baseline characteristics of study area**

**Description**	**Intervention areas**	**Control areas**	**Total**
Households	16802	18659	35461
Population	137781	145543	283324
Number of areas	20	20	40
Public health facilities	28	27	55
Number of LHWs/CHWs	165	141	306
Number of TBAs	85	93	178

The intervention package, consisting of awareness creation about positive maternal and newborn health care practices at household level such as importance of seeking antenatal care, adequate nutrition during pregnancy and lactation, skilled birth attendance (ANC, early initiation of breastfeeding, delayed bathing and recognition of danger signs that warrant for early referrals, was developed in collaboration with the Aga Khan Health Services, Pakistan. The practices were promoted though community mobilization and education strategy that included formation of Community Health Committee (CHC) and group education sessions using flip charts and videos.

The LHWs and CHWs in the intervention areas were provided with enhanced trainings on causes of perinatal and newborn mortality and risky maternal and newborn care practices and were expected to transmit the knowledge to the families to avoid such practices. They received training in delivering the intervention package through standardized workshops including hands on practice on use of specific Information Education and Communication (IEC) materials developed for this purpose. The recently modified and simplified integrated management of neonatal and childhood illnesses (IMNCI) based system formed the basis of screening, recognition of danger signs and referral. The CHWs delivered interventions in LHW uncovered areas within the intervention areas.

Control areas continued to receive the routine services of governmental and non-governmental organizations in the area. But the public health care facilities of both intervention and control areas received similar competency based trainings in stabilization and early referral of sick newborns for secondary care. Since the existing primary care staffs at the level of BHUs and RHCs were already well versed in other essentials of maternal and antenatal care such as nutritional counseling, contraceptive advice & provision and iron-folate supplementation, our intervention focused on augmenting additional aspects of perinatal and newborn care.

### The intervention package

The LHWs and CHWs received trainings on IMNCI-based training package. They were also given orientation about the purpose of the project and how can they facilitate group education session by using flip charts and videos. The community-based health education sessions were introduced targeting local communities to sensitize them regarding maternal, perinatal and newborn health issues.

In addition to this, Two days training workshops were organized to train 85 TBAs in intervention areas on “Clean Delivery Practices” at nearest health facilities. The UNICEF manual of (DAI) training was adopted for this training. However, the LHWs and CHWs promoted skilled birth attendance as policy of AKHSP and health department through group sessions.

The intervention package was implemented through monthly household visits, one-to-one counseling sessions with pregnant women and video sessions in communities. Additionally, LHWs and CHWs in the intervention areas were asked to record information about home visits, newborn illnesses, referrals, live births and deaths on special format designed for this project.

There was no additional human resources were added to roll out the intervention except two community mobilizers hired and trained by the project. The mobilizers (one male and one female) assisted the LHWs and CHWs in identifying community members form community health committees (CHCs) in their respective catchment population in the intervention areas. The aim of forming these CHCs was promoting perinatal and newborn care in their areas and get community influencer’s support on key household target practices. The CHCs formed under the project were likely to continue after the end of the project because these were part of the remit of existing outreach health care workers.

For community mobilization and education, two types of tools were used one group session by use of flip charts and group session by use of video. Participants were invited from all Muhallas (Sub-geographical distribution of the village population) to attend the session, facilitated by LHW/CHW to organize the session. Separate sessions were organized for males and females. One session per area was organized on quarterly basis in local school or LHW health house or CHW household within intervention areas. The sessions were attended by women of reproductive age, adolescent girls, fathers, mothers and fathers in law and mothers in law.

### Data collection

To evaluate the trends and estimate the impact of the intervention package; quarterly surveillance system was established and baseline as well as end line cross-sectional surveys were conducted through an independent data collection teams to collect information on vital statistics such as live births, perinatal and neonatal deaths, maternal and newborn care practices from both the intervention and control areas. The study was approved by the ethical review committee of Aga Khan University Karachi, Pakistan.

We hired eight data collectors and two team leaders with education levels of graduation and masters respectively. They received three days training in the areas of data collection techniques, ethical issues and on data collection tools. Two independent project surveillance teams identified major pregnancy outcomes through quarterly surveillance visits. These independent data collection teams identified pregnancies, live births, stillbirths and neonatal deaths through quarterly surveillance visits to each household for the period of sixteen months. These were cross checked against available information collected by a separate team on all births, deaths and newborn referrals within the public and private (AKHSP) health facilities. In addition information was also cross checked with LHW program data collected by LHWs as part of their official information systems on births, deaths and referrals.

Verbal autopsies of still births and neonatal deaths were conducted by Research Medical Officer or a trained field staff member. We used WHO standard verbal autopsy questionnaire for collecting data on cause of deaths. The verbal autopsy tool was used by AKU in previous similar studies conducted to ascertain cause of death in rural Sindh research sites of Hala and Kotdiji [17]. The verbal autopsies were conducted for classification of cause-specific deaths.

### Data management and statistical analysis

Visual Fox-Pro was used for designing the databases, the data entry software and the procedures for data quality assurance. Data entry employed range and consistency checks and skips to minimize entry of erroneous data. Statistical package for social sciences (SPSS Version 16) was used to analyze data. The data analysis included descriptive and inferential analyses. The descriptive analysis was run to understand and describe the survey participants and their characteristics. In the second stage of analysis inferential statistics using regression analysis was done to drive the odds ratios (OR), Confidence intervals (CI) and P value. DiD model was used to assess the impact of interventions, which were defined as the changes in the outcome variables in intervention areas minus those experienced in control areas. In univariate analysis, this method involves simply making proper subtractions.

## Results

Baseline study areas characteristics such as number of households and population and the number of health care workers and number health care facilities were comparable in both intervention and control areas (Table 1). Baseline information on births and deaths like live births, stillbirths and perinatal deaths were comparable at baseline between intervention and control areas and there was no significant different in the mortality indicators between both areas (Table 2).

**Table 2:** **Comparison of pre and post intervention in vital characteristics**

**Characteristics**	**Pre intervention**			**Post intervention**		
	**Intervention areas**	**Control areas**	**p-value**	**Intervention areas**	**Control areas**	**p-value**
Live births	846	854		833	842	
Total births	871	875		849	863	
Still births	25	21		16	21	
Perinatal deaths	41	45		30	48	
Early Neonatal deaths	16	24		14	27	
Neonatal deaths	22	34		19	33	
Infant deaths	26	37		10	27	
**Perinatal Mortality Rate per 1000 total births**	**47.1**	**51.4**	**0.76**	**35.3**	**55.6**	**0.02**
**Early Neonatal Mortality Rate per 1000 live births**	**19.0**	**28.1**	**0.24**	**16.8**	**32.1**	**0.02**
**Neonatal Mortality Rate per 1000 live births**	**26.0**	**39.8**	**0.13**	**22.8**	**39.2**	**0.03**

The community-based interventions were mainly implemented through LHWs/CHWs in support of project community mobilizers. A total 165 LHWs and CHWs were trained with additional curriculum on essential newborn care. The counseling skills of LHWs and CHWs in intervention areas were improved. LHWs and CHWs in the intervention areas established 110 community health committees (CHCs) in all 90 villages with 816 members. A total 218 community sessions were organized using flip charts and 121 video sessions, with the help of CHCs for local community on perinatal and newborn care health issues. In total, around 6764 participants attended these sessions. Of these participants, 6353 were females and 411 were males. LHWs and CHWs in the intervention areas visited 2636 (83%) households of 3150 planned newborn assessment visits. They also examined 72 sick newborns who were identified at household level. It is important to note that CHWs were also present in the control areas but were not provided any additional training.

Highly significant proportion of women in the intervention areas than in control areas reported antenatal care visits during pregnancy (92% vs 76%, p < .001), uptake of TT vaccination during pregnancy (67% vs 47%, p < .001) and delivery conducted at health facility (85% vs 71%, p < .001) at end line. Moreover, the reported practices of colostrum administration as first feed (83% vs 64%, p < .001), initiation of breast feeding within one hour after birth (55% vs 40%, p < .001) and EPI vaccination for newborns was comparatively higher in intervention areas than in control areas. Women in intervention areas were less likely to report practice of traditional cord application (51% vs 71%, p < .001) than the women in control areas and first bath given to newborn baby within one hour after birth (15% vs 43%, p < .001). DiD estimates were highest for first bath to newborn within 1 hours after birth (−26%) and TT vaccination during pregnancy (17%), while were modest for cord application (−15%), initiation of breastfeeding within 1 hour after birth (12%), EPI vaccination for newborns (12%), colostrum administration as first feed (11%), delivery conducted at health facility (11%) and seeking ANC during pregnancy (10%) (Table 3).

**Table 3:** **Pre and post intervention changes in maternal and newborn care practices using DID estimates**

**Indicators**	**Intervention**				**Control**				**DID**
	**Pre**	**Post**	**Change**	**P-value**	**Pre**	**Post**	**Change**	**P-value**	**(a-b)**
	**N = 322**	**N = 316**	**(a)**		**N = 386**	**N = 361**	**(b)**		**(%)**
	**n (%)**	**n (%)**	**(%)**		**n (%)**	**n (%)**	**(%)**		
**Pregnant women**									
- Seek ANC during pregnancy	245 (76)	290 (92)	16	<.001	270 (70)	274 (76)	6	.076	10
- TT vaccination during pregnancy	155 (48)	212 (67)	19	<.001	174 (45)	170 (47)	2	.602	17
- Delivery conducted at health facility	229 (71)	269 (85)	14	<.001	262 (68)	257 (71)	3	.355	11
**Newborn care practices**									
- Cord application	238 (74)	161 (51)	−23	<.001	305 (79)	259 (71)	−8	.028	−15
- First bath to newborn within 1 hour	116 (36)	47 (15)	−21	<.001	147 (38)	154 (43)	5	.206	−26
- Colostrum administration as first feed	225 (70)	262 (83)	13	<.001	239 (62)	234 (64)	2	.435	11
- Initiation of breastfeed within 1 hour	135 (42)	175 (55)	13	<.001	151 (39)	144 (40)	1	.839	12
- EPI vaccination for newborns	225 (70)	279 (88)	18	<.001	243 (63)	250 (69)	6	.085	12

Consequently, during the intervention period reported stillbirths and perinatal deaths were higher in control areas compared with intervention areas (21 reported stillbirths and 48 perinatal deaths in control areas vs 16 and 30 in intervention areas). Neonatal mortality remained unchanged in control areas during study periods (39.8 per 1000 live births vs 39.2), while in intervention areas it seemed to decrease (from 26 per 1000 live births to 22.8). (Table 2).

Overall mortality indicators for the intervention and control areas at baseline were comparable. There were significant reductions in mortality in intervention areas as compared to control areas from baseline in Perinatal Mortality Rate (from 47.1 to 35.3 per 1000 births, OR 0.62; 95% CI: 0.56-0.69; P 0.02) and neonatal mortality rates (from 26.0 to 22.8 per 1000 live births, 0.58; 95% CI: 0.48-0.68; P 0.03) (Table 2).

The majority of stillbirths were related to asphyxia conditions as they occurred in the intra-partum period. The causes of death among neonates were prematurity (36.3%), neonatal sepsis (25.8%), birth asphyxia (18.5%), tetanus (6.5%), birth injury (3.2%) congenital abnormality (2.4%) and unexplained deaths (7.3%) (Table 4).

**Table 4:** **Classification of cause specific mortality among neonates**

**Causes of neonatal deaths**	**Number of deaths**	**Percent**
Prematurity	45	36.3
Sepsis	32	25.8
Birth asphyxia	23	18.5
Tetanus	8	6.5
Birth injury	4	3.2
Congenital abnormality	3	2.4
Unexplained deaths	9	7.3

## Discussion

Our study shows that selected community-based maternal, and newborn care interventions delivered through outreach health workers were associated with reduction of perinatal and neonatal mortality in the intervention areas. Moreover, key healthy maternal and newborn care practices were improved in intervention areas than in control areas and other studies have shown reduction of newborn mortality and morbidity outcomes by implementing community based interventions globally [6-10].

Evidence generated by our group also showed reduction in neonatal and perinatal mortality in rural areas of southern rural district of Pakistan by implementing community based interventions delivered through mainly LHWs and TBAs [6,9]. The reduction in the mortality was potentially achieved mainly due to adoption of healthy and quitting harmful household maternal and newborn health practices such as seeking of ANC and not applying traditional cord applications. Main causes of neonatal deaths in our study area were prematurity, neonatal sepsis and birth asphyxia. A study from neighboring country India also showed similar causes of neonatal mortality [18].

Improvements in maternal and newborn care household practices were reported in intervention areas as compared to control areas. Seeking antenatal care during last pregnancy was almost similar both in intervention and control areas at the time of baseline but at endline it increased by 16% i.e. from 76% to 92%. Cord application is identified as risk factor for neonatal infections [13,15,19,20], in the baseline it was very high in both intervention and control areas but decreased in the intervention areas as compared to control areas (74% & 79). The biggest changes occurred in practices related to discarding application of traditional cord application and delaying of first bath to newborn within 1 hour of birth at the endline in intervention areas. Research from neighboring country India also showed positive impact of training health workers on household newborn are practices [21].

At the baseline introduction of colostrum as first feed was low in both intervention areas and control areas (70% vs 62%), but it increased up-to 13% in intervention areas and only 2% in control areas at the endline, many studies from India and other South Asian countries have indicated that women commonly avoided giving colostrum to newborns due to their misconceptions that it is harmful for the health of newborns [22-25]. Moreover, early initiation of breastfeeding within 1 hour of birth was increased in intervention areas compared with control areas (55% vs 40%). Other studies also confirmed success in promoting early initiation of breastfeeding through introduction awareness raising interventions such as counseling of mothers about the benefits of early initiations of breastfeeding [6,7,24].

It was also encouraging to note that the TT vaccination rates during pregnancy increased by 19%, i.e. from 48% at baseline to 67% at endline in intervention areas. Facility birth rates were almost similar at baseline in both intervention and control areas (71% vs 68%), but it increased up-to 14% in intervention areas and only 3% in control areas at endline. The reason of low facility births in our study area might be due to difficult geographical terrain and lack of 24/7 availability of skilled birth providers and other facilities in public and NGO run health care facilities.

The recent Pakistan Demographic and Health Survey (PDHS) 2012–13 showed no change in reduction in neonatal mortality since last PDHS survey conducted in 2006–07. This underscores the fact that the burden of preventable neonatal mortality will not reduce unless evidence-based maternal and newborn care interventions are scaled up universally in rural and remote areas. Moreover, it is pertinent to note that NMR in both areas was significantly less than national NMR of 55/1,000, live births likely due to existing vast infrastructure of health services by AKHSP in addition to the public sector health facilities.

The LHWs program in rural Pakistan is considered to be the backbone of primary health care including maternal and child health and covers approximately 60% of the rural population [26-29]. Moreover, in the LHW uncovered areas CHWs such as AKHSP workers and other volunteer mechanisms are available. These human resources available at community level should be tapped in to roll out low tech preventive and health promotion interventions related to maternal and newborn health. Our study findings indicate that LHWs and CHWs can play a key role in implementing interventions that improve maternal and newborn health and survival specially in hard to reach mountainous areas. The randomized controlled trials evaluating impact of community level interventions in rural Pakistan and other neighboring countries support the scale-up of preventive and promotive maternal and newborn care interventions through community health workers [6,21,30-33].

The results of this study should be read while keeping in view some of the limitations. As part of this study surveillance system was in place to collect information on births and deaths (still births and neonatal deaths) but it was not prospective. Hence there may a chance of missing to collect information of any birth or death. We used strict measures for data quality assurance to collect robust data and data was verified with other sources such as government and private sector records. The mechanism of supervision was introduced to validate the data. In addition to this, errors may have arisen in ascertainment and misclassification of stillbirths and early neonatal deaths. The time period for intervention exposure was limited due to funding constrains. The geography of the area and climate must be considered before the planning of any project as these factors can influence the project operations especially in mountainous areas. We did not meet the deadlines to organize master trainers training workshop due to geographical and weather conditions that limited travel possibilities of maters trainers to the study area. Hence any activities planned in such settings must have some flexibility factored in and contingency planning done to meet the challenges of weather conditions prevailing in the study area.

One of the major strengths of our study was the fact that the intervention package was implemented largely through existing public sector health workers and locally trained AKHSP CHWs rather than the project paid employees. Hence the interventions can likely be feasible and sustainable through local health systems. The design of our project was robust and underwent ethical clearance and data was collected through an independent data collection system.

## Conclusions

The data indicate that the implementation of a set of low tech community-based interventions within the local health system settings in a mountainous region of Pakistan helped reduce the burden of perinatal and neonatal mortality. Policy level attention is required to scale up evidence based and effective interventions at community level through existing outreach health workers to make the dent in the stagnant neonatal mortality in Pakistan.
